# An improved synchronous reference frame current control strategy for a photovoltaic grid-connected inverter under unbalanced and nonlinear load conditions

**DOI:** 10.1371/journal.pone.0164856

**Published:** 2017-02-13

**Authors:** Amirreza Naderipour, Abdullah Asuhaimi Mohd Zin, Mohd Hafiz Bin Habibuddin, Mohammad Reza Miveh, Josep M. Guerrero

**Affiliations:** 1 Faculty of Electrical Engineering, Universiti Teknologi Malaysia, UTM Skudai, Johor, Malaysia; 2 Institute of Energy Technology, Aalborg University, Aalborg East, Denmark; West Virginia University, UNITED STATES

## Abstract

In recent years, renewable energy sources have been considered the most encouraging resources for grid and off-grid power generation. This paper presents an improved current control strategy for a three-phase photovoltaic grid-connected inverter (GCI) under unbalanced and nonlinear load conditions. It is challenging to suppress the harmonic content in the output current below a pre-set value in the GCI. It is also difficult to compensate for unbalanced loads even when the grid is under disruption due to total harmonic distortion (THD) and unbalanced loads. The primary advantage and objective of this method is to effectively compensate for the harmonic current content of the grid current and microgrid without the use of any compensation devices, such as active and passive filters. This method leads to a very low THD in both the GCI currents and the current exchanged with the grid. The control approach is designed to control the active and reactive power and harmonic current compensation, and it also corrects the system unbalance. The proposed control method features the synchronous reference frame (SRF) method. Simulation results are presented to demonstrate the effective performance of the proposed method.

## Introduction

Among the various renewable energy sources (RESs), photovoltaics (PVs) are the most promising environmental friendly and fastest growing clean and renewable energy source [1,2]. The RESs are connected to the utility network or microgrid (MG) by an interface converter. An MG is a local grid composed of distributed generators (DGs), energy storage systems and loads and can operate in both grid-connected [3] and island modes [4]. Power quality problems are a specific concern with MGs because distortion harmonic sources can represent a high proportion of the total loads or nonlinear loads (NLLs) in small-scale systems [5]. The main limitation associated with MGs occurs when exchanging the current from the grid to the MG [6–8]; this exchange is considered a source of harmonic distortion in a grid-connected inverter (GCI) [9]. To improve the power quality in MGs, several approaches have been proposed [10]. Installing passive filters (PFs) in the appropriate locations, preferably close to the harmonic generator, can lead to trapping of the harmonic currents near the source and can reduce their distribution throughout the other parts of the system [11]. Active power filters (APFs) have been proven as a flexible solution for compensating the harmonic distortion caused by various NLLs in power distribution systems [12]. Hybrid compensation (HC) has the advantages of both passive and active power filters for the improvement of power quality problems [13]. Traditionally, the GCIs used in MGs connected to the main grid have behaved as current sources [14]. The GCI controller should be able to correct an unbalanced system and cancel the main harmonics to meet the waveform quality requirements of the local loads and MGs [9]. The primary goal of a power-electronic interface inverter is to control the power injection [15]. However, compensation for power quality problems, such as current harmonics, can be achieved through appropriate control strategies. Consequently, the control of DGs must be improved to meet the requirements when connected to the grid [16].

In the literature [9,17–24], several methods have been presented to control the DGs in terms of a current harmonic compensator. The methods in [17] and [9] were proposed to compensate for current harmonics in grid-connected MGs. The current controller proposed in [17] uses the synchronous reference frame (SRF) and is composed of a proportional–integral (PI) controller and a repetitive controller (RC). The other study [9] proposed a cascaded current and voltage control strategy for the interface converter in MGs. M. Hamzeh et al. [19] proposed a control strategy that includes a harmonic impedance controller and a multi-proportional resonant controller. Additionally, one researcher presented a control method for a multi-bus MV MG under unbalanced and nonlinear load conditions [24]. Several controllers, namely, PI controllers implemented in the *dq* frame as well (also constituting an SRF), a resonant controller, a PI controller implemented in the *abc* frame, and a dead-beat (DB) predictive controller, were proposed in [23]. In 2013 Savaghebi et al. published a paper in which they proposed methods to compensate for the voltage unbalance at the RER terminal; the power quality at the point of common coupling (PCC) is usually the main concern due to sensitive loads that may be connected [25]. Mohamed Abbes et al. [26] proposed a control strategy for a three-level, neutral point clamped (NPC) voltage source converter. Two current controllers were designed to achieve grid current control.

Unfortunately, traditional APFs have several drawbacks, including higher cost, larger size, higher power switch count, and complex control algorithms and interface circuits to compensate for unbalanced and nonlinear loads.

Due to the aforementioned issues, this study presents a new inverter control method for harmonic compensation. The proposed control strategy consists of an SRF method, which is proposed to control power injection to the grid, provide harmonic current compensation and correct the unbalanced system. The focus of the present paper is the reduction of total harmonic distortion (THD) in the current flowing between the PCC and MG. Furthermore, simulation studies are presented, discussed and analysed. The main proposed control methods are as follows:

- voltage and current controllers;
- active and reactive power controllers;
- harmonic current compensation;
- current unbalance compensation.

This paper is organized as follows. The proposed control scheme for the DG GCI is discussed in Section II. In this section, details of the entire control structure, including the active and reactive power control unit and harmonic current compensation unit, are explained. Simulation results with three case studies are presented in Section III. Finally, conclusions are presented in Section IV.

### The proposed control method

**Fig 1:**
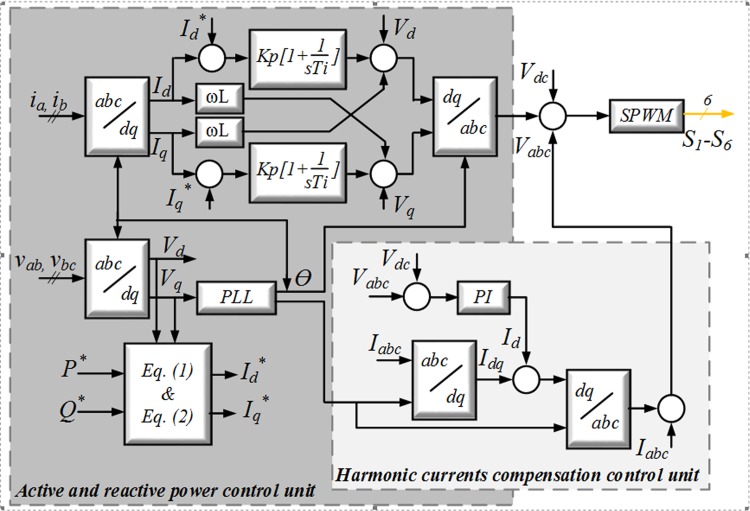
Block diagram of the GCI’s proposed control method.

To enhance grid and MG current quality, an advanced current control method for the GCI is presented. The proposed method contains two units: the active and reactive power control unit and the harmonic current compensation unit. Fig 1 presents a schematic block diagram of the proposed control strategy for the GCI. This block diagram is applicable for main and remaining harmonic compensation, reactive power supply for distortion sources and system unbalance correction.

### Active and reactive power control unit

**Fig 2:**
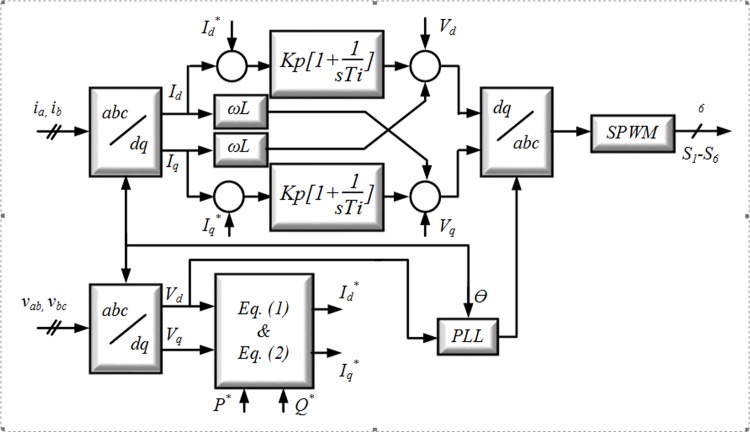
Block diagram of the control active and reactive powers.

In the grid-connected power control mode, essentially all the available power that can be achieved from the microturbine (MT) [27], fuel cell (FC) [28] and PV is delivered to the grid [29]. Furthermore, compensation of the reactive power is possible. A block diagram of the control arrangement for the grid-connected control mode is shown in Fig 2.

Certain controllers, namely, the PI controllers, are implemented in the *dq* frame (also called the SRF) to adjust the grid currents in the *dq*-synchronous frame. The well-known SRF method can be used for control of the APF and GCI, and it also can be designed as in [30]. This method uses a reference frame transformation module, *abc*→*dq*. The *dq* transformation can be used to convert the three phase currents injected by the inverter into three constant DC components defined as the direct, quadrature and zero components: *I*_*d*_, *I*_*q*_ and *I*_*0*_, respectively. In general, three phase voltages and currents are transformed into *dq0* coordinates by the Park transformation, as shown by the matrix [L]:

$$\left[\begin{array}{c} u_{d}\\ u_{q}\\ u_{0} \end{array}\right]=\left[L\right]\left[\begin{array}{c} u_{A}\\ u_{B}\\ u_{C} \end{array}\right]\;and\;\left[\begin{array}{c} i_{d}\\ i_{q}\\ i_{0} \end{array}\right]=\left[L\right]\left[\begin{array}{c} i_{A}\\ i_{B}\\ i_{C} \end{array}\right]$$ (1)

$$\left[L\right]=\sqrt{\frac{2}{3}}\left[\begin{array}{ccc} \sin\alpha & \sin\left(\alpha -\frac{2\pi }{3}\right) & \sin\left(\alpha +\frac{2\pi }{3}\right)\\ \cos\alpha & \cos\left(\alpha -\frac{2\pi }{3}\right) & \cos\left(\alpha +\frac{2\pi }{3}\right)\\ \frac{1}{\sqrt{2}} & \frac{1}{\sqrt{2}} & \frac{1}{\sqrt{2}} \end{array}\right]$$ (2)

The phase angles of the voltage and current signals are set as a reference current, which achieves the SRF while I^*^_d_ *= 0*. Fig 1 shows the current controller of interest [31]. The sinusoidal pulse width modulation (SPWM) voltage frame is guaranteed. The voltage reference and design phase-locked loop (PLL) synchronize the inverter with the grid. Thus, I^*^_d_ and I^*^_q_ as the reference currents in the *dq* transform are re-calculated as follows:

The reference currents in the *dq-axis*, I^*^_d_ and I^*^_q_, can be obtained from the following relations:

$$P=V_{d}I_{d}+V_{q}I_{q}\overset{V_{q=0}}{\to }I_{d}^{*}=\frac{P^{*}}{V_{d}}$$ (3)

$$Q=V_{d}I_{q}-V_{q}I_{d}\overset{V_{q=0}}{\to }I_{q}^{*}=\frac{Q^{*}}{V_{d}}$$ (4)

where *V*_*d*_ and *V*_*q*_ are the grid voltages in the *dq* transform. Furthermore, the inverter is able to deliver P^*^ and Q^*^, which are the reference active and reactive power, respectively.

The simplified active and reactive powers are calculated as:

$$\begin{array}{l} P=V_{d}I_{d}\\ Q=V_{d}I_{q} \end{array}$$ (5)

Eq 5 shows that although the system voltage is constant (*V*_*d*_), the currents of *dq* control the active and reactive powers.

The real power injection from the GCI is controlled by the reference signal of I^*^_d_, whereas the reactive power is set to zero (I^*^_q_ *= 0*). The reference current I^*^_d_ is extracted from dynamic analysis of the DC-link capacitor. A constant DC voltage across the capacitor shows that the DG matches the power. The equation is:

$$\frac{d}{dt}V_{DC}^{2}=\frac{2}{C}(P_{in}-P_{out})$$ (6)

The DC-DC converter controls the power input (*P*_*in*_) to the capacitor to generate the maximum DG output power. To keep the inverter output voltage constant, the inverter controls the capacitor output power (*P*_*out*_). The reference current is extracted from the difference between *P*_*in*_ and *P*_*out*_ using the PI controller.

$$I_{d}^{*}=\frac{1}{V_{d}}(K_{p}(P_{in}-P_{out})+K_{I}\int \left(P_{in}-P_{out})dt\right)$$ (7)

From these parameters, the command voltages V^*^_d_ and V^*^_q_ for the inverter gates SPWM can be acquired using:

$$\begin{array}{l} V_{d}^{*}=K_{p}(I_{d}^{*}-I_{d})+K_{I}\int \left(I_{d}^{*}-I_{d})dt-\omega L_{f}I_{q}+V_{d}\right)\\ V_{q}^{*}=K_{p}(I_{q}^{*}-I_{q})+K_{I}\int \left(I_{q}^{*}-I_{q})dt-\omega L_{f}I_{d}+V_{q}\right) \end{array}$$ (8)

The command voltages are directed to the inverter for PWM. The control strategy applied to the interface converter usually includes two cascaded loops. An external voltage loop controls the DC-link voltage, and a fast internal current loop regulates the grid current. The DC-link voltage in this structure is controlled by the essential output power, which is the reference for the active current controller. Typically, the *dq* control methods are associated with PI controllers because they have satisfactory behaviour when regulating *DC* variables [32]. Eq 9 gives the matrix transfer function in *dq* coordinates:

$$G_{PI}^{\left(dq\right)}(s)=\left[\begin{array}{cc} K_{p}+\frac{K_{i}}{s} & 0\\ 0 & K_{p}+\frac{K_{i}}{s} \end{array}\right]$$ (9)

where *K*_*p*_ and *K*_*i*_ are the proportional and integral gain of the controller, respectively.

**Fig 3:**
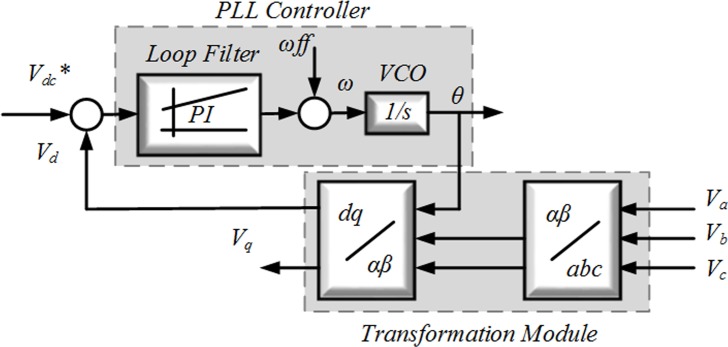
PLL structure of the three phase.

To provide the phase information of the grid voltage, the PLL technique [33] can be used, which is required to generate the current reference *i*_*ref*_ and is also used to maintain the GCI synchronism with the grid. To create the three current references, a PLL system has been proposed in which the error of each is directed into the controller, and the corresponding measured current can be compared. Regarding the switches in the GCI, the switching of the output of these controllers is noteworthy. When three PI controllers are used, the modulator is essential for creating the duty cycles for the SPWM pattern. The PLL schematic is illustrated in Fig 3.

In this synchronization structure, *ɷ*_*ff*_ and *ɷ* are the fixed frequency and the estimated grid frequency, respectively. As depicted in Fig 3, the value of the rated frequency normally consists of a feed-forward *ɷ*_*ff*_ to improve the dynamics of the phase estimation *ɵ*, which is obtained by integrating *ɷ*.

### Harmonic current compensation control unit

In the control unit for the compensation of harmonic currents, a three-phase stationary rotating frame is converted to a synchronous rotating frame via Park’s transformation. This transform for the active and reactive components of the load voltage and current [34] is illustrated in (10):

$$\left[\begin{array}{c} i_{d}\\ i_{q} \end{array}\right]=\left[\begin{array}{ccc} \sin\theta & \sin\left(\theta -\frac{2\pi }{3}\right) & \sin\left(\theta +\frac{2\pi }{3}\right)\\ \cos\theta & \cos\left(\theta -\frac{2\pi }{3}\right) & \cos\left(\theta +\frac{2\pi }{3}\right) \end{array}\right]$$ (10)

$$i_{d}={\bar{I}}_{{}_{d}}+{\tilde{I}}_{{}_{d}}$$ (11)

$$i_{q}={\bar{I}}_{{}_{q}}+{\tilde{I}}_{{}_{q}}$$ (12)

The active and reactive components of the current are decomposed into the *dq* components in (11) and (12).

The current source generates the fundamental component of the *d-axis* of the load and the filter DC component associated with the strategy of harmonic compensation.

The steady-state error of the inverter’s DC component is eliminated by the PI controller to keep the voltage across the capacitor constant. The error voltage is computed by comparing the DC capacitor voltage with the reference voltage. Moreover, to regulate the capacitor under dynamic conditions and mitigate the steady-state error, the output of the PI controller is modified. The PLL performance has a significant effect on the *dq* transform of the output signal. In a PLL, the rotating reference frame (*ɷt*) is set as a fundamental component. Application of the inverse of Park’s transformation is represented in (13) through (15). The *dq* rotating frame is converted back to a three-phase stationery frame:

$$i_{sa}^{*}=i_{d}\sin\left(\omega t\right)+\cos\left(\omega t\right)$$ (13)

$$i_{sb}^{*}=i_{d}\sin\left(\omega t-\frac{2\pi }{3}\right)+\cos\left(\omega t-\frac{2\pi }{3}\right)$$ (14)

$$i_{sc}^{*}=i_{d}\sin\left(\omega t+\frac{2\pi }{3}\right)+\cos\left(\omega t+\frac{2\pi }{3}\right)$$ (15)

This theory is only applicable to the three-phase system. Because the harmonic current compensation controller addresses DC quantities, its control unit is entirely stable. However, there is a time delay in filtering the DC quantities due to the instantaneous computation of quantities [35]. The extracted reference signal is then used to trigger the gate driver controller, which will allow the inverter to introduce the desired compensation current into the system.

### Simulation results

**Fig 4:**
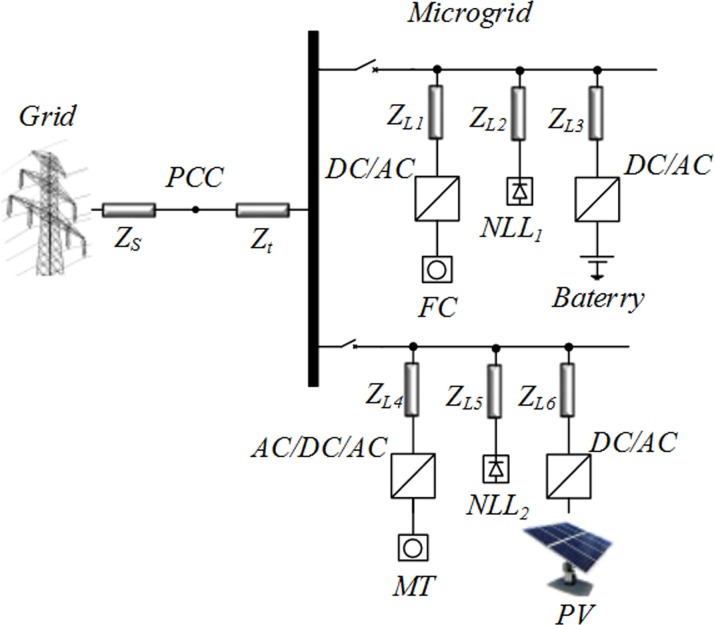
Study system configuration with DGs and distorted loads.

In a basic MG architecture (Fig 4), the system is assumed to be radial with several feeders and a collection of NLLs. To demonstrate the effectiveness of the proposed control strategy, the system in Fig 4 was simulated in MATLAB/Simulink.

This MG includes three DGs, namely, the PV, MT and FC, which are connected to the grid by the power electronic interface. The proposed control methods are applied to the PV; however, the FC is connected to the grid by the ordinary interface converter without the control strategy. The MT has a frequency of 1,500 Hz, which is similar to a normal generator, but its output voltage has a frequency of 1,500 Hz. Therefore, the effective voltage of the output phase of this 220 V MT has a frequency of 1,500 Hz, but this source cannot be connected to a power system with a frequency of 50 Hz. For this purpose, the input voltage must first be rectified using a diode rectifier, and the maximum output voltage of the rectifier will be 530 V. Then, the level of the output voltage can be raised to 750 V using a boost convertor connected to an inverter transformer. Thereafter, the DC voltage is applied to the interface converter, which is controlled by the SRF controller.

Furthermore, the FC has an output of 50 kW at 625 VDC and is connected to the grid by an ordinary AC/DC convertor. Another side of this system consists of two NLLs, such as the three unbalanced single-phase diode rectifiers and the three-phase diode rectifier, which produced the distorted waveform.

The PFs are distributive and are connected near the unbalanced NLLs and DGs in the system; an APF is connected at the PCC. In operation, the PF is used to compensate for the major harmonics. The APF is located at the upstream position to correct the unbalance of the system and to remove the remaining harmonics.

The parameters of the three-phase power line [13], load/DGs and control method parameters can be found in Tables 1, 2 and 3, respectively. In this system, the voltage is assumed to be sinusoidal.

**Table 1:** Power Line Parameters.

	Z_L1_	Z_L2_	Z_L3_	Z_L4_	Z_L5_	Z_L6_	L_t_	L_s_
R (Ω)	0.263	0.524	0.781	0.134	0.280	0.134	-	-
L (mH)	0.631	1.33	1.92	0.312	0.652	0.312	0.165	0.015

**Table 2:** Load/DG Parameters.

Load/DGs	Parameters	Values
MT	Inverter switching frequency	4 kHz
	Inverter resistance	4 Ω
	Inverter capacitance	5 μF
	DC-link voltage	545 V
PV	Inverter switching frequency	4 kHz
	Inverter resistance	0.2 mΩ
	Inverter capacitance	5 μF
	DC-link voltage	675 V
FC	Inverter resistance	0.1 mΩ
	Inverter capacitance	0.1 μF
	DC-link voltage	625 V
Battery	Inverter resistance	4 Ω
	Inverter capacitance	5 μF
	DC-link voltage	750 V
Rating of NLL 1	RL	30 kW, 10 kVAr
Rating of NLL 2	Resistor	0.3 Ω

**Table 3:** Control Parameters.

Controller	Parameter value
*V_DC_ (V)*	688
Proportional gain (*K_p_*)	0.4
Integral gain (*K_i_*)	10
Fundamental frequency (*H_z_*)	50
*V_abc_* (*V*) and *V_abc DG_* (*V*)	220
*I_abc_ (A)*	210
*I_injected_ (A)*	47

**Fig 5:**
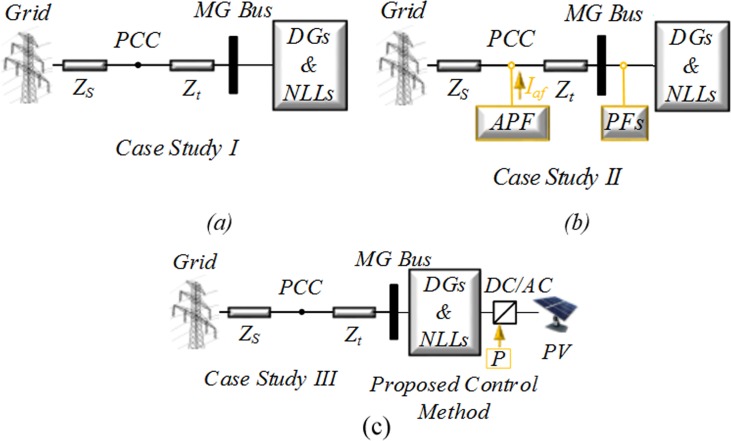
Schematics of a system and an MG: (a) without any compensation devices; (b) with active and passive filters; (c) using just the proposed control method on the PV.

In the simulation, three case studies are taken into account. The configurations of the three case studies are illustrated in Fig 5.

Case study I: Without any compensation devices.

Case study II: With an APF and distributed PFs.

Case study III: Without any compensation devices, such as APF and PFs, and with only the proposed control method on the PV.

Fig 5 shows the three studied system configurations. Fig 5(A) consist of DG units and NLLs connected to the grid without any compensation devices. As shown in Fig 5(B), each PF is connected near the DGs and NLLs and designed to eliminate main harmonics and supply reactive power to the MG.

### Case study I

**Fig 6:**
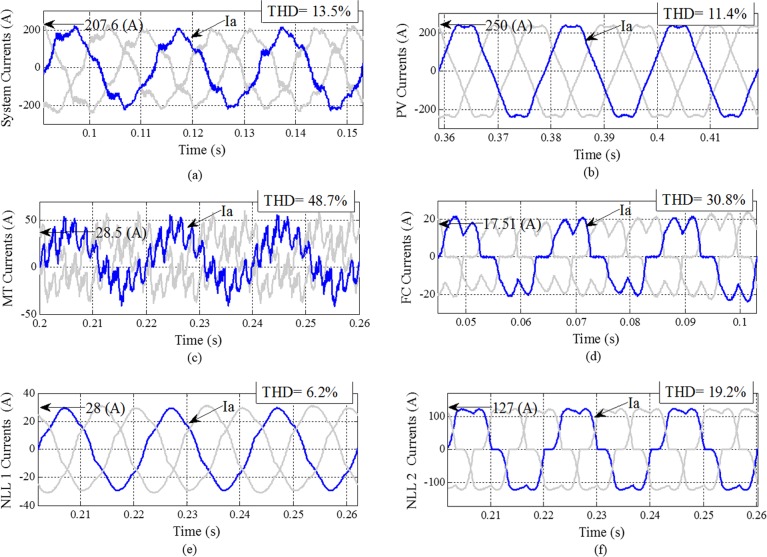
System DG unit currents and NLL current waveforms with THD without any compensation: (a) system currents; (b) PV currents; (c) MT currents; (d) FC currents; (e) NLL 1 currents; (f) NLL 2 currents.

The currents of DGs and NLLs, equipped without any compensation devices and the proposed control strategy, are shown in Fig 6. The GCI and NLLs make the system current nonlinear and unbalanced and also require the injection of harmonic currents into the MG and grid. The waveforms of the three-phase output currents and the distortion currents of the DGs under NLL conditions are depicted in Fig 6(A). All output currents of the DGs and NLLs have an effect on the system currents and produce a distortion waveform with 13.5% THD. The output currents of three DGs are highly distorted with sharp spikes. Fig 6(B) shows the PV array output current. The PV is connected to the MG through a GCI, which produces the distorted waveforms with 11.04% THD. Furthermore, the MT and FC are connected to the system through power electronic converters. Normally, the GCI discussed in the paper is considered as a harmonic current source; in this case, the parallel compensation system is the best choice. Thus, the DGs and NLLs may be considered to be current sources in terms of harmonic distortion. Fig 6(E) and 6(F) illustrate the NLL current waveform and the associated harmonic spectra.

### Case study II

**Fig 7:**
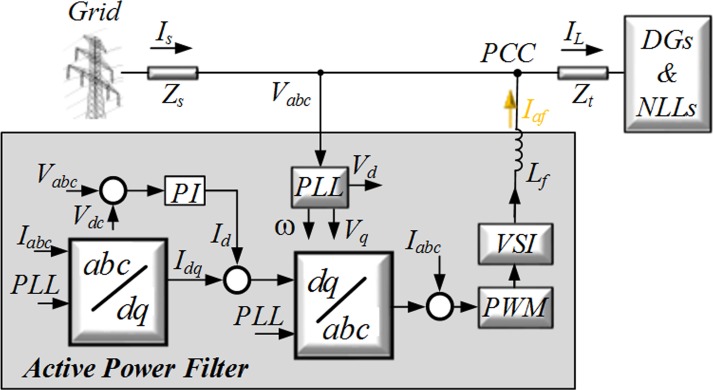
Block diagram of the control system for the APF.

This case study consists of an APF and a distributed PF. Each PF is connected to DGs and NLLs and is designed to eliminate the main harmonics and supply reactive power for NLLs, while the APF is responsible for the correction of the system unbalance and the removal of the remaining harmonics. Fig 7 shows the *dq* current control block diagram of the APF.

**Fig 8:**
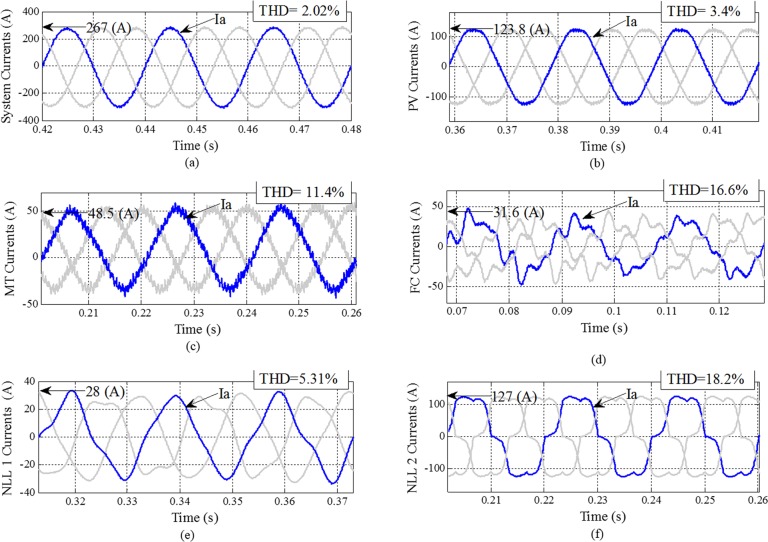
System DG unit current waveforms with THD after compensation with active and passive filters: (a) system currents; (b) PV currents, (c) MT currents; (d) FC currents; (e) NLL 1 currents; (f) NLL 2 currents.

A system waveform using active and passive filter compensation is shown in Fig 8. An active filter is connected to the system; the system harmonics and unbalanced current are compensated. Fig 8(A) provides the results obtained from the simulation after the connection of dedicated compensation devices that can reduce the THD in the system from 13.5% to 2.02%. Comparing the waveforms in Figs 6(B)–6(F) and 8(B)–8(F), the effects of the PFs and APF can be clearly identified.

After the active filter is connected, the source current becomes balanced and sinusoidal. Without any compensation, the system current THD is 13.56%; after compensation, the THD is reduced to 2.02%.

### Case study III

**Fig 9:**
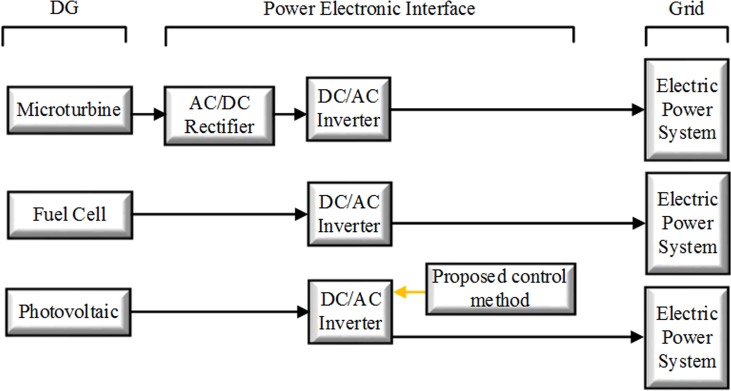
Block diagram of the power electronic interface configuration.

This case study indicates improved power quality with the absence of compensation devices in the MG. The main contribution of this study is the harmonic current compensation between the PCC and the MG. The compensated system currents are explained in this subsection. The proposed control method can be applied to the GCI of the PV. Fig 9 shows a block diagram of the power electronic interface configuration for the MT, FC and PV with installation of the proposed control method.

**Fig 10:**
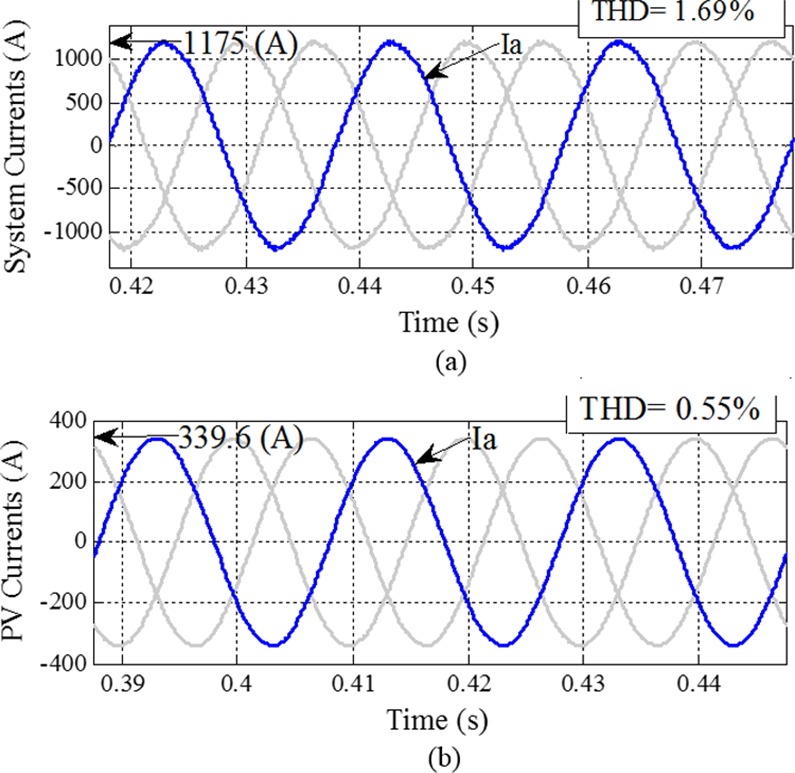
System and DG unit current waveforms with THD without any compensation devices except for the proposed control method on the PV: (a) system currents; (b) PV currents.

Before the GCI with proposed control method is switched in, the system current contains harmonics and is unbalanced. The proposed control method is effective in correcting the distortion. Fig 10(A) and 10(B) show the effective compensation values of the harmonic current for the system and PV, respectively. This case study shows that the proposed control method can compensate for the current system (PCC) and DGs in the absence of power compensation devices and an APF. The output currents of phase ‘a’ in the PV and their harmonic spectra, without the harmonic compensation loop, are depicted in Fig 6. The output currents of phase ‘a’ in the PV are distorted because of the connection of the three-phase NLL. Both negative and zero sequence harmonics, consisting of the 3rd, 5th, 7th, 9th, 11th and 13th harmonics, can cause power quality problems in the grid-connected MG. The output current quality for PV can be improved using the proposed harmonic compensator loop that consists of six modules, tuned at the 3rd, 5th, 7th, 9th, 11th and 13th harmonic frequencies. For performance comparison, the output current of phase ‘a’ in the DGs and their harmonic spectra, with the proposed harmonic controller, are shown in Fig 10. When comparing the output current waveforms in phase ‘a’, it is notable that by employing the proposed control method, the 3rd, 5th, 7th, 9th, 11th and 13th harmonics in the current are substantially suppressed.

When all of the loads and DGs are connected, the THD in the system without any compensation is 13.5% (Fig 6(A)). As shown in Fig 10(A), the THD is reduced to 1.69% at the PCC with the proposed control method. The simulation results of Fig 10(A) and 10(B) show the performance of the proposed control method for compensating the distorted waveform of Fig 6(A) and 6(B). The effects of the proposed control method can be clearly observed by comparing the bold values in Table 4 (between case study I and case study III). Thus, this approach is verified to be capable of meeting IEEE 519–1992 recommended harmonic standard limits. The currents and the THD value of the system in all case studies are given in Table 4. Full names corresponding to nomenclature are shown in S1 File.

**Table 4:** Current and THD results.

Identifier	Case Study I			Case Study II		Case Study III	
	Current (A)	THD %		Current (A)	THD %	Current (A)	THD %
System	207.6		**13.56**	267	2.02	1175	**1.69**
PV	123.8		**11.46**	123.8	3.4	339.6	**0.55**
MT	28.52		48.72	48.5	11.46	758.6	48.72
FC	17.51		30.87	31.66	16.68	70.64	12.40
NLL 1	28		6.26	28	5.31	19.8	6.40
NLL 2	127		19.25	127	18.02	87.01	19.44

## Conclusions

This study proposes a new control strategy for harmonic current compensation for DG interface converters in an MG. The proposed control method consists of the SRF control method. When nonlinear, unbalanced loads and DGs are connected to the grid, the proposed strategy significantly and simultaneously improves the THD of the interface converter for DGs and the grid current.

In the proposed method, the harmonic currents of the NLLs and DGs in the MG and at the PCC are completely compensated. The proposed control is responsible for controlling the active and reactive power injection to the grid; it is also responsible for compensating for the harmonic currents due to the unbalanced load. The presented simulation results show that the PCC harmonic currents due to unbalanced loads, NLLs and DGs are compensated to the desired value. This strategy can be used for single-phase and three-phase systems. The simulation results verify the feasibility and effectiveness of the newly designed control method for a grid-connected converter in an MG.

## Supporting information

S1 File Nomenclature. (PDF)

## References

[1] Wei J, Bai D, Yang L. Polymer Photovoltaic Cells with Rhenium Oxide as Anode Interlayer. PLoS One. Public Library of Science; 2015;10: e0133725 10.1371/journal.pone.0133725 26226439 PMC4520519

[2] Xu G, Moulema P, Ge L, Song H, Yu W. A Unified Framework for Secured Energy Resource Management in Smart Grid Smart Grid. CRC Press; 2016 pp. 73–96.

[3] Humada AM, Hojabri M, Sulaiman MH Bin, Hamada HM, Ahmed MN. Photovoltaic Grid-Connected Modeling and Characterization Based on Experimental Results. PLoS One. Public Library of Science; 2016;11: e0152766 10.1371/journal.pone.0152766 27035575 PMC4817980

[4] Samrat NH, Ahmad N, Choudhury IA, Taha Z. Technical Study of a Standalone Photovoltaic–Wind Energy Based Hybrid Power Supply Systems for Island Electrification in Malaysia. PLoS One. Public Library of Science; 2015;10: e0130678 10.1371/journal.pone.0130678 26121032 PMC4488286

[5] Golovanov N, Lazaroiu GC, Roscia M, Zaninelli D. Power quality assessment in small scale renewable energy sources supplying distribution systems. Energies. Multidisciplinary Digital Publishing Institute; 2013;6: 634–645.

[6] Song H, Rawat DB, Jeschke S, Brecher C. Cyber-Physical Systems: Foundations, Principles and Applications. Morgan Kaufmann; 2016.

[7] Dou H, Qi Y, Wei W, Song H. A two-time-scale load balancing framework for minimizing electricity bills of internet data centers. Pers Ubiquitous Comput. Springer; 2016;20: 681–693.

[8] Naderipour A, Zin AAM, Bin Habibuddin MH. IMPROVED POWER-FLOW CONTROL SCHEME FOR A GRID-CONNECTED MICROGRID. Electron WORLD. ST JOHN PATRICK PUBL 6 LAURENCE POUNTNEY HILL, LONDON, EC4R OBL, ENGLAND; 2016;122: 42–45.

[9] Zhong Q-C, Hornik T. Cascaded current–voltage control to improve the power quality for a grid-connected inverter with a local load. Ind Electron IEEE Trans. IEEE; 2013;60: 1344–1355.

[10] Zin AAM, Naderipour A, Habibuddin MH, Guerrero JM. Harmonic currents compensator GCI at the microgrid. Electron Lett. IET; 2016;

[11] Das JC. Passive filters-potentialities and limitations. Pulp and Paper Industry Technical Conference, 2003 Conference Record of the 2003 Annual. IEEE; 2003. pp. 187–197.

[12] Akagi H. New trends in active filters for power conditioning. Ind Appl IEEE Trans. IEEE; 1996;32: 1312–1322.

[13] Chen Z, Blaabjerg F, Pedersen JK. Hybrid compensation arrangement in dispersed generation systems. Power Deliv IEEE Trans. IEEE; 2005;20: 1719–1727.

[14] Guerrero JM, Vasquez JC, Matas J, Castilla M, de Vicuña LG. Control strategy for flexible microgrid based on parallel line-interactive UPS systems. Ind Electron IEEE Trans. IEEE; 2009;56: 726–736.

[15] Jiang D, Zhang P, Lv Z, Song H. Energy-efficient Multi-constraint Routing Algorithm with Load Balancing for Smart City Applications. IEEE Internet of Things Journal. 2016 p. 1.

[16] Blaabjerg F, Teodorescu R, Liserre M, Timbus A V. Overview of control and grid synchronization for distributed power generation systems. Ind Electron IEEE Trans. IEEE; 2006;53: 1398–1409.

[17] Trinh Q-N, Lee H-H. An Enhanced Grid Current Compensator for Grid-Connected Distributed Generation Under Nonlinear Loads and Grid Voltage Distortions. IEEE; 2014;

[18] Cordeschi N, Shojafar M, Baccarelli E. Energy-saving self-configuring networked data centers. Comput Networks. Elsevier; 2013;57: 3479–3491.

[19] Hamzeh M, Karimi H, Mokhtari H. Harmonic and Negative-Sequence Current Control in an Islanded Multi-Bus MV Microgrid. Smart Grid, IEEE Trans. IEEE; 2014;5: 167–176.

[20] Jara AJ, Sun Y, Song H, Bie R, Genooud D, Bocchi Y. Internet of Things for Cultural Heritage of Smart Cities and Smart Regions. Advanced Information Networking and Applications Workshops (WAINA), 2015 IEEE 29th International Conference on. IEEE; 2015. pp. 668–675.

[21] Shojafar M, Cordeschi N, Amendola D, Baccarelli E. Energy-saving adaptive computing and traffic engineering for real-time-service data centers. 2015 IEEE International Conference on Communication Workshop (ICCW). IEEE; 2015. pp. 1800–1806.

[22] Yang X, He X, Yu W, Lin J, Li R, Yang Q, et al Towards a Low-Cost Remote Memory Attestation for the Smart Grid. Sensors. Multidisciplinary Digital Publishing Institute; 2015;15: 20799–20824. 10.3390/s150820799 26307998 PMC4570448

[23] Timbus A, Liserre M, Teodorescu R, Rodriguez P, Blaabjerg F. Evaluation of current controllers for distributed power generation systems. Power Electron IEEE Trans. IEEE; 2009;24: 654–664.

[24] Hamzeh M, Karimi H, Mokhtari H. A new control strategy for a multi-bus MV microgrid under unbalanced conditions. Power Syst IEEE Trans. IEEE; 2012;27: 2225–2232.

[25] Savaghebi M, Jalilian A, Vasquez JC, Guerrero JM. Autonomous voltage unbalance compensation in an islanded droop-controlled microgrid. Ind Electron IEEE Trans. IEEE; 2013;60: 1390–1402.

[26] Abbes M, Belhadj J. New control method of a robust NPC converter for renewable energy sources grid connection. Electr Power Syst Res. Elsevier; 2012;88: 52–63.

[27] Ranjbar M, Mohaghegh S, Salehifar M, Ebrahimirad H, Ghaleh A. Power electronic interface in a 70 kW microturbine-based distributed generation. Power Electronics, Drive Systems and Technologies Conference (PEDSTC), 2011 2nd. IEEE; 2011. pp. 111–116.

[28] Khunjar WO, Sahin A, West AC, Chandran K, Banta S. Biomass production from electricity using ammonia as an electron carrier in a reverse microbial fuel cell. PLoS One. Public Library of Science; 2012;7: e44846 10.1371/journal.pone.0044846 23028643 PMC3446996

[29] Hossain MA, Xu Y, Peshek TJ, Ji L, Abramson AR, French RH. Microinverter thermal performance in the real-world: Measurements and modeling. PLoS One. Public Library of Science; 2015;10: e0131279 10.1371/journal.pone.0131279 26147339 PMC4493157

[30] Teodorescu R, Blaabjerg F. Flexible control of small wind turbines with grid failure detection operating in stand-alone and grid-connected mode. Power Electron IEEE Trans. IEEE; 2004;19: 1323–1332.

[31] Jou H-L, Chiang W-J, Wu J-C. A simplified control method for the grid-connected inverter with the function of islanding detection. Power Electron IEEE Trans. IEEE; 2008;23: 2775–2783.

[32] Twining E, Holmes DG. Grid current regulation of a three-phase voltage source inverter with an LCL input filter. Power Electron IEEE Trans. IEEE; 2003;18: 888–895.

[33] Arruda LN, Silva SM. PLL structures for utility connected systems. Industry Applications Conference, 2001 Thirty-Sixth IAS Annual Meeting Conference Record of the 2001 IEEE. IEEE; 2001. pp. 2655–2660.

[34] Salam Z, Tan PC, Jusoh A. Harmonics mitigation using active power filter: A technological review. Elektrika. Faculty of Electrical Engineering; 2006;8: 17–26.

[35] Shi P, Zhang Y, Chadli M, Agarwal RK. Mixed H-Infinity and Passive Filtering for Discrete Fuzzy Neural Networks With Stochastic Jumps and Time Delays. IEEE; 2015; 10.1109/TNNLS.2015.2425962 25974953

