# Supplementary material for: An improved synchronous reference frame current control strategy for a photovoltaic grid-connected inverter under unbalanced and nonlinear load conditions
Source: PLoS One. 2017 Feb 13;12(2):e0164856. doi: 10.1371/journal.pone.0164856 (PMC5305230; doi:10.1371/journal.pone.0164856)
Supplement: S1 File — (PDF) [file pone.0164856.s001.pdf]

Nomenclature

u	Grid voltage
P	Real power injected
Q	Reactive power injected
ɵ	Phase estimation
C	Capacitor
ϖ	Frequency of rotation of the reference frame in rad/s
L	Transformation matrix
i_{ref}	Reference current
ω_{ff}	Frequency of rotation of the reference feed forward
V_{DC}	DC-link capacitor voltage
P_{in}	Input power
P_{out}	Output power
PI	Proportional integral
K_p and K_I	PI controller constants
i_a, i_b and i_c	Three-phase load currents
$\overline{I_d}$ and $\overline{I_q}$	Fundamental active and reactive current components
$\widetilde{I_d}$ and $\widetilde{I_q}$	Harmonic active and reactive current components
i_{sa}^*, i_{sb}^* and i_{sc}^*	Extracted reference current signal
v_d and v_q dq	Voltages at the point of common coupling
I_d and I_q dq	Currents at the point of common coupling
I_d^* *and* I_q^* dq	Reference currents
